# Extra-Uterine Pregnancy: A Clinical and Operative Study

**Published:** 1900-03

**Authors:** 


**Extra-Uterine Pregnancy: a Clinical and Operative Study.** By JOHN W. TAYLOR. Pp. x., 205. London: H. K. Lewis. 1899.

The surgeon will find this book a very good guide upon a subject that requires all the light that can be thrown on it. The chapters on Diagnosis and Treatment are the best we have ever read; their chief merit consists in the very practical way in which the subject is treated. We congratulate the author on the very successful series of cases with which he has illustrated his work.

